# A method to estimate prey density from single-camera images: A case study with chinstrap penguins and Antarctic krill

**DOI:** 10.1371/journal.pone.0303633

**Published:** 2024-07-09

**Authors:** Victoria R. Hermanson, George R. Cutter, Jefferson T. Hinke, Matthew Dawkins, George M. Watters

**Affiliations:** 1 Antarctic Ecosystem Research Division, Southwest Fisheries Science Center, National Marine Fisheries Service, National Oceanic and Atmospheric Administration, La Jolla, CA, United States of America; 2 Kitware Inc., Clifton Park, NY, United States of America; University of Tasmania, AUSTRALIA

## Abstract

Estimating the densities of marine prey observed in animal-borne video loggers when encountered by foraging predators represents an important challenge for understanding predator-prey interactions in the marine environment. We used video images collected during the foraging trip of one chinstrap penguin (*Pygoscelis antarcticus*) from Cape Shirreff, Livingston Island, Antarctica to develop a novel approach for estimating the density of Antarctic krill (*Euphausia superba*) encountered during foraging activities. Using the open-source Video and Image Analytics for a Marine Environment (VIAME), we trained a neural network model to identify video frames containing krill. Our image classifier has an overall accuracy of 73%, with a positive predictive value of 83% for prediction of frames containing krill. We then developed a method to estimate the volume of water imaged, thus the density (N·m^-3^) of krill, in the 2-dimensional images. The method is based on the maximum range from the camera where krill remain visibly resolvable and assumes that mean krill length is known, and that the distribution of orientation angles of krill is uniform. From 1,932 images identified as containing krill, we manually identified a subset of 124 images from across the video record that contained resolvable and unresolvable krill necessary to estimate the resolvable range and imaged volume for the video sensor. Krill swarm density encountered by the penguins ranged from 2 to 307 krill·m^-3^ and mean density of krill was 48 krill·m^-3^ (sd = 61 krill·m^-3^). Mean krill biomass density was 25 g·m^-3^. Our frame-level image classifier model and krill density estimation method provide a new approach to efficiently process video-logger data and estimate krill density from 2D imagery, providing key information on prey aggregations that may affect predator foraging performance. The approach should be directly applicable to other marine predators feeding on aggregations of prey.

## Introduction

The use of animal-borne instrumentation has expanded studies on the physiology [1] and ecology [2] of free-ranging marine predators. A variety of telemetry devices have enabled the detailed characterization of diving energetics and behaviors [3, 4], movement and migration patterns [5, 6], habitat partitioning [7, 8], and identification of essential marine habitats [9, 10]. Accelerometers mounted on the head or jaw [11–13], ingested stomach temperature probes [14], and movement data [15], (but see [16]) have been used to estimate feeding rates. Direct observation of predation events, prey, and foraging habitats have been done for large animals such as seals [17–20] and whales [21–25], but assessing predator responses to changes in prey availability remains difficult in the marine environment, particularly for smaller animals such as penguins [26–30]. While there are studies that have observed penguins striking at prey [12, 31, 32], in this work we quantify the prey field and prey availability, which may influence feeding behaviors and success.

Miniaturization of digital cameras has allowed first-person views of underwater behaviors in a suite of marine species [33–35], including penguins [23, 27, 36–39]. Importantly, these image-based methods offer direct observations of the interactions between predator and prey and provide the potential for novel inference on key ecological interactions. Images and data derived from animal-borne video loggers may provide a useful step toward understanding predator responses to variation in prey density. For example, video data allow assessments of the frequency of encounters with prey and estimates of predator foraging rate within prey patches [40, 41]. However, estimating the density of prey from two-dimensional (2D) images obtained from animal-borne cameras represents a significant challenge for two main reasons. First, visual analysis and manual annotation of video imagery to identify prey encounters is slow and inefficient, requires subjective interpretation, and may be affected by observer error and bias. Second, when prey are visible in imagery, counts of individuals within the image can provide a relative index of prey abundance, but estimating the concentration of prey (volumetric density) additionally requires estimating the imaged volume. To overcome these challenges, we trained an automated detection algorithm to classify video content at the frame-level and developed a method to estimate the volumetric density observed in 2D imagery of prey encountered by a foraging predator.

Machine learning algorithms are increasingly used in the ecological studies to automate detection of features of interest in imagery [42, 43]. Frame-level classifier models can be trained to identify images that contain characteristics indicative of behavior, locations, or the environment. For example, in animal-borne video data from an air-breathing marine predator, the characteristics of images obtained at the surface are distinct from those obtained underwater, and images obtained during open-water diving are different from those obtained during encounters with prey aggregations. Classifier models can be trained to differentiate such characteristics [44, 45] and identify frames with prey encounters for further analysis. We leverage automated image processing to alleviate the burden of time spent manually processing hours of video imagery. The open-source computer vision platform VIAME (Video and Image Analytics for the Marine Environment [46]; available from https://www.viametoolkit.org/) provides a flexible and powerful tool for image annotation, detector training, and image analysis. We used VIAME to develop a classifier model to automate identification of images containing krill based on full frame-level content.

Several approaches can be used to estimate three-dimensional (3D) scene structure from single camera 2D imagery. An approach developed to remove haze from images, to estimate light transmission, and enhance underwater imagery using the dark channel prior [47, 48] produces a depth map of the scene that is useful for characterizing topography and relative distances among scene elements. Most methods for 3D estimation from single images produce depth maps [49] with relative distances to objects that must be scaled based on known sizes of scene elements at known ranges to estimate actual coordinates or sizes.

**Fig 1:**
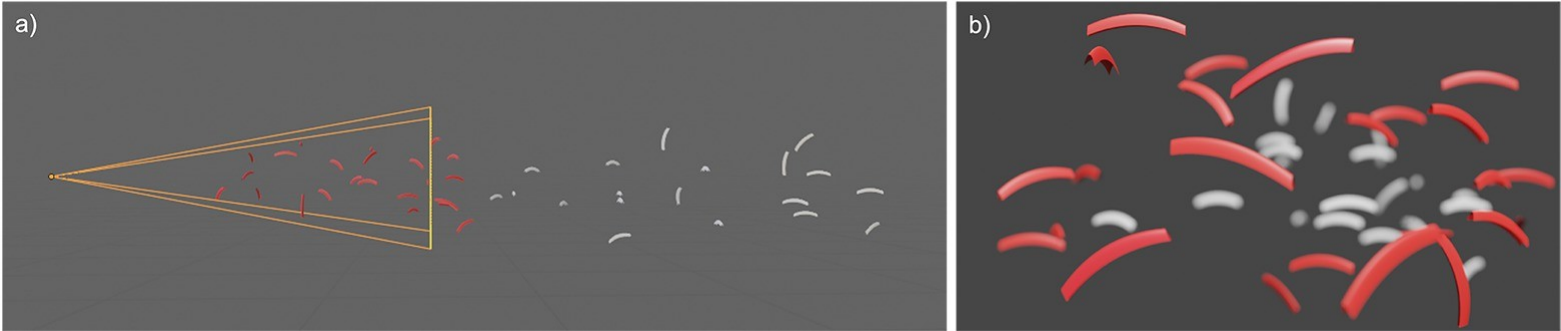
Two-panel view of the resolvable range of the camera. a) side view and b) camera view of targets with simulated depth of field effects.

Other single-camera, single image approaches for estimating distance to imaged targets use object focus methods, also known as the depth from defocus [50], to reconstruct 3D scenes from individual 2D images without the assistance of additional sensors or multiple cameras [51–54]. Depth-from-defocus methods assume that imaged targets within a 2D scene can be unresolved due to large ranges from the imaging system, limits of resolution, or because of relative motion of the object and camera. Assuming that targets have a known length, combined with a distinction between resolvable (containing discernible visible features) and unresolvable targets (lacking distinctive features or out of focus because of range and not as a result of motion blur) in the image can enable the estimation of the imaged range, therefore reconstructing a 3D scene (Fig 1).

Here, we used animal-borne video imagery to first train a frame-level image classifier with a convolutional neural network model to identify images that represent basic penguin behaviors including surfacing/diving, surface resting, underwater transit/swimming, and prey encounters. Based on a subset of images containing krill, we then implement a method conceptually similar to the approaches for estimating single-image depth of field and depth from defocus [53–55] to estimate the volumetric density (N·m^-3^) of krill swarms encountered by foraging chinstrap penguins.

## Materials and methods

### Instrumentation

We collected video imagery of the interactions between chinstrap penguins and their principal prey, Antarctic krill, from Cape Shirreff, Livingston Island (60.79°W, 62.46°S) in the northern Antarctic Peninsula region. Videos were collected in February 2018 and December 2019 using Little Leonardo DVL400M028 (52 mm × 20 mm × 11mm, 15 g) and DVL400M065 (61 mm × 21 mm × 15 mm, 29 g) digital video loggers (DVLs). The DVLs recorded color imagery using ambient light at 30 frames per second (fps). Field-of-view angles were 31ᐤ horizontal by 24ᐤ vertical and the image frame size was 1280 by 960 pixels (pix). Videos were recorded continuously for 5 to 8 hours (separated by 30-minute intervals when exported) until memory capacity was full or the battery was exhausted. Each penguin was also instrumented with a time depth recorder (TDR; Lotek LAT1800FP, 36x13x11 mm, 9 g) to record dive depth. Instrument attachment and recovery methods were described previously [40].

### Ethical approval

All animal handling procedures and research protocols are approved by the Southwest Fisheries Science Center/Pacific Islands Fisheries Science Institutional Animal Care and Use Committee (# SWPI 2020–01). All field research activities are permitted under the U.S. Antarctic Conservation Act (Permit #2017–012).

### Automated image analysis

**Fig 2:**
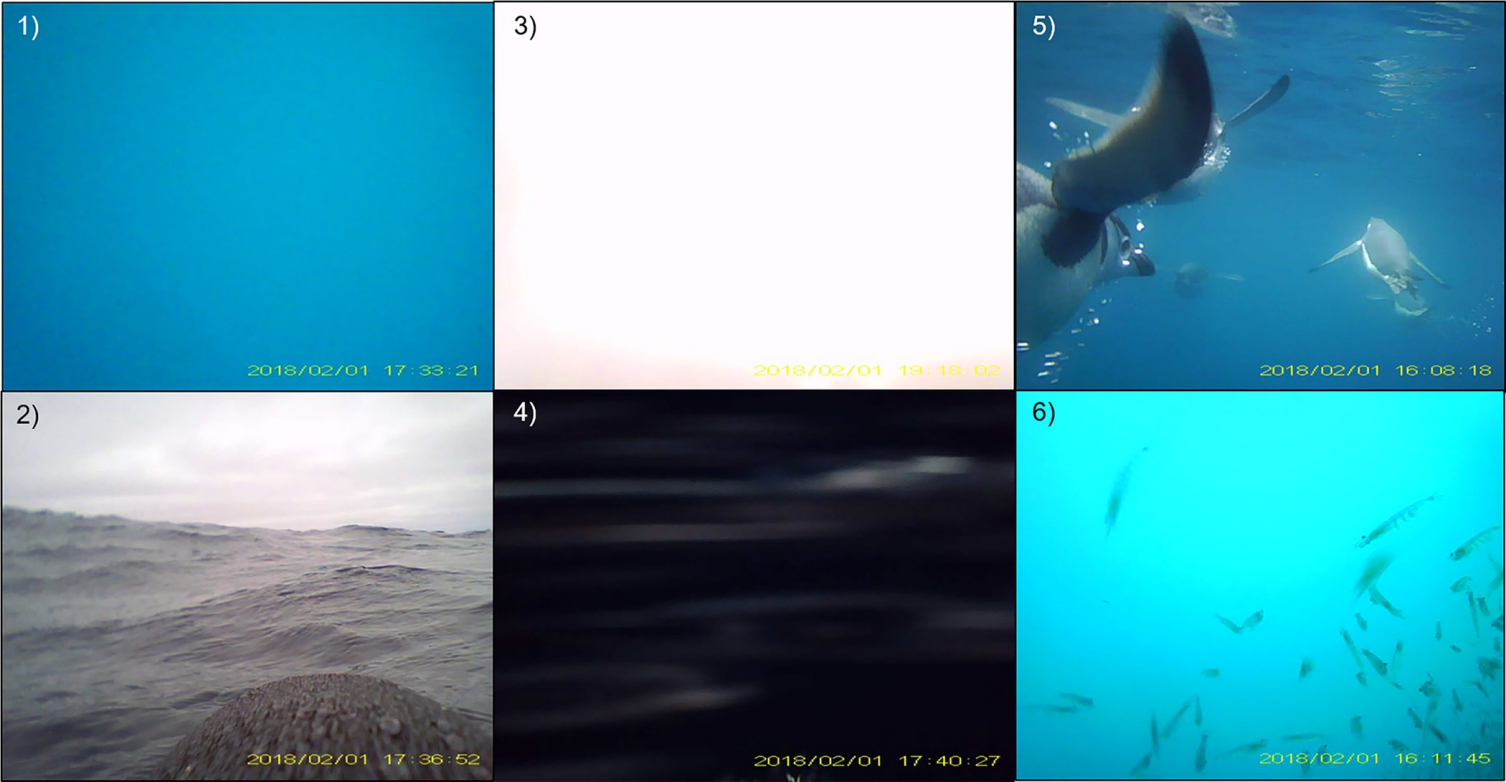
Example images of the frame-level classes used for model training. The five classes represent images from 1) open water representing underwater transit/swimming, 2) surface, representing resting on the surface, 3) bright, during surfacing or diving, 4) dark, during surfacing or diving, 5) penguin presence, 6) krill presence.

We used the imagery from both 2018 and 2019 to train a frame-level image classifier model. We used VIAME (version 0.19.4) to train an EfficientNet convolutional neural network [56] to classify the principal frame-level content of video images. The model was trained on a 1,638 image subset using a five-class scheme (Table 1). Each of the classes represent the frame-level visual content associated with behaviors or observed conditions recorded during foraging dives that represent: 1) **open water**, indicative of underwater swimming; 2) **surface**, indicative of surface activities such as breathing and resting during intervals between dives; 3) **bright or dark**, very bright or dark conditions indicative of passing through the air-water interface during diving or surfacing events; 4) **penguin presence** (while swimming underwater) indicative of group association; 5) **krill presence**, indicative of prey encounters (Fig 2). The number of images per class in the training set ranged from 2 to 815 (Table 1).

**Table 1:** Number of training and test images for each class. Each class is representing the principle visual content associated with penguin behaviors and observed conditions while foraging. Training and test images were taken from video collected in February 2018 and December 2019.

Training label	Training Images	Test Images
Open water	815	320
Krill presence	147	262
Surface	553	324
Penguin presence	2	21
Bright or dark	121	73

### Model performance evaluation

Model performance was evaluated with a confusion matrix using the R (R Core Team 2020) package ‘caret’ [57]. The trained model was tested on a subset of 1,000 new images. In conjunction, manual annotations (a truth set) were completed on the test subset to use for the confusion matrix evaluation.

Confusion matrix metrics relating true classes to model-predicted classes were represented using counts of true positives, false positives, true negatives, and false negatives from model predictions. True Positives (TP) represent correct model predicted class matching the known, analyst-designated (“truth”) class of an image. True Negatives (TN) are all images that the model correctly identifies as not containing the class; False Positives (FP) are cases where the predicted class does not represent the true class. False Negatives (FN) are images predicted as negative for a class when they should represent the true class. For each class we calculated:

$$accuracy:\;A=\frac{TP+TN}{TP+FP+FN},$$

$$precision\;(positive\;predicted\;value):\;P=\frac{TP}{TP+FP},$$

$$recall\;(sensitivity):\;R=\frac{TP}{TP+FN},$$

$$specificity=\frac{TN}{TN+FP},$$

negtivepredictivevalue:NPV=(specificity*(1−prevalence))/(((1−sensitivity)*prevalence)+((specificity*(1−prevalence)),

$$balanced\;accuracy=\frac{sensitivity+specificity}{2},$$

$$prevalence=\frac{N_{class}}{N_{total}},\;where\;N_{total}\;is\;the\;count\;by\;class\;and\;N_{total}\;is\;the\;total\;number\;of\;images\;in\;the\;truth\;set,\;and\;the$$

$$F1\;score:\;F1=\frac{2*(P*R)}{P+R}.$$

[58–60]

These metrics were calculated to evaluate the performance of the classifier model. After model performance was evaluated, our classifier model was used to detect frames containing from a new image set (1.5 hours of video sampled at 5 fps collected in February 2018) to be used for prey density estimation.

### Estimating prey density from 2D imagery

The images used to estimate krill density were manually selected from a set of images that the EfficientNet classifier model predicted to be krill from 1.5 hours of video sampled at 5 fps collected in February 2018. From these we selected a subset of 124 images where motion blur was minimal and the image included targets distributed from near to far, thus allowing classification of resolvable and unresolvable krill (Fig 1).

**Fig 3:**
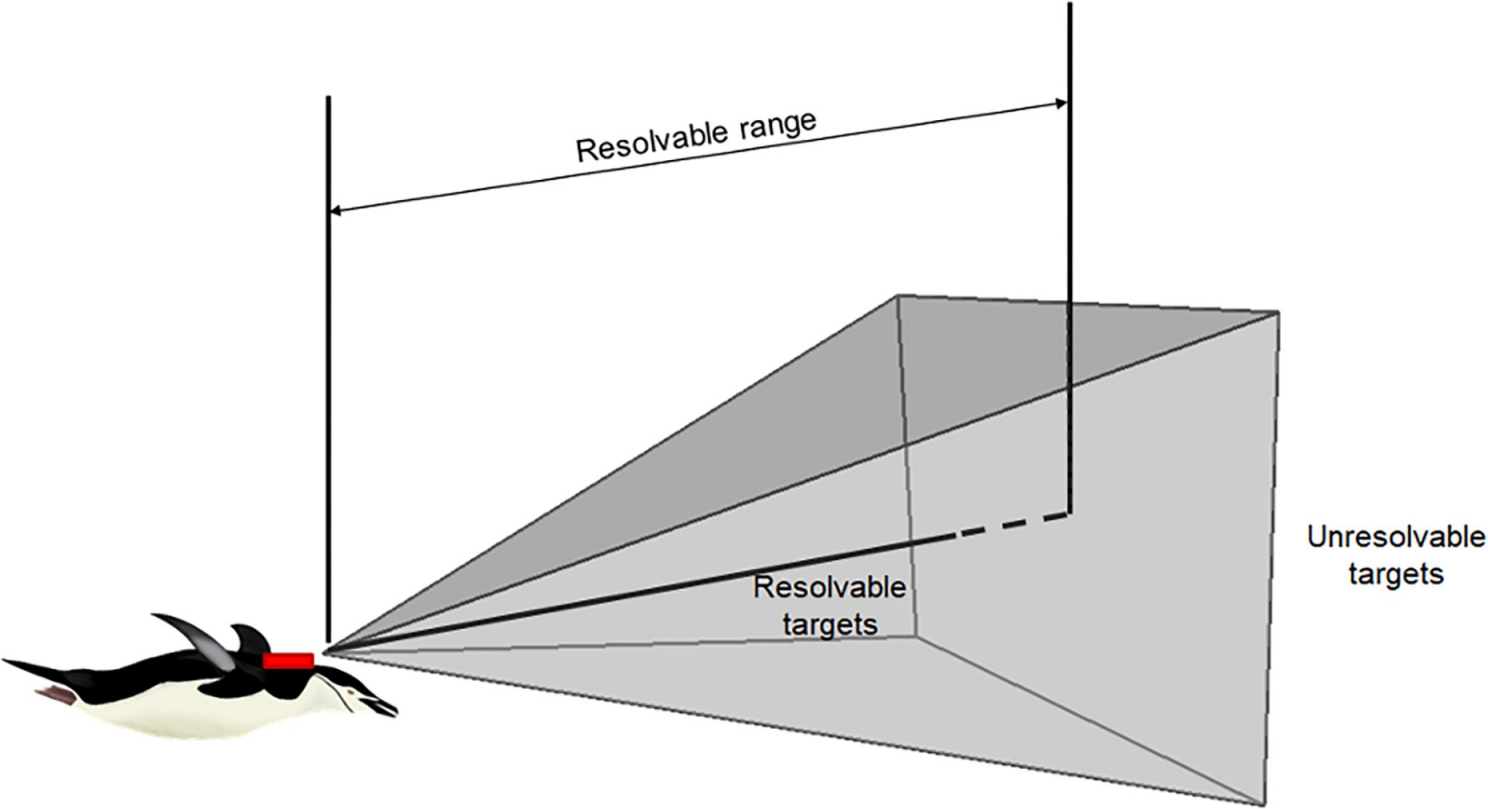
Conceptual image for estimating the resolvable range of the camera and the imaged volume. Only resolvable targets are included within the resolvable range. Imaged volume was estimated from the resolvable range (r_res_) and the field-of-view of the camera lens.

The density of krill in an image was estimated by assuming the imaged volume of a photograph to be represented by a triangular pyramid (Fig 3). We define the “resolvable range” (r_res_) as the maximum range within which imaged targets (krill) are clearly identifiable. By relating the imaged length of targets at the limit of the resolvable range to known krill lengths, and using information on the dimensions of the image, we estimated the physical range to these targets to solve for the imaged volume. Counts of resolvable krill within the imaged volume were then converted to density estimates. Camera and lens calibrations were not done, but distortions were negligible based on visual inspection of images. For example, objects like other penguins had consistent appearances in various locations (e.g. center vs. corner) and the horizon appeared as a straight line in images from surface intervals.

### Resolvable range and imaged volume

To estimate the densities of krill encountered during a penguin’s foraging event, we first estimate the volume of water sampled by the images by estimating the resolvable range (Fig 3). Estimating resolvable range minimally requires an estimate of the length of krill (in pixels) at the distance representing the limit of the imaged volume and an estimate of the true length (in mm) of the imaged targets.

**Fig 4:**
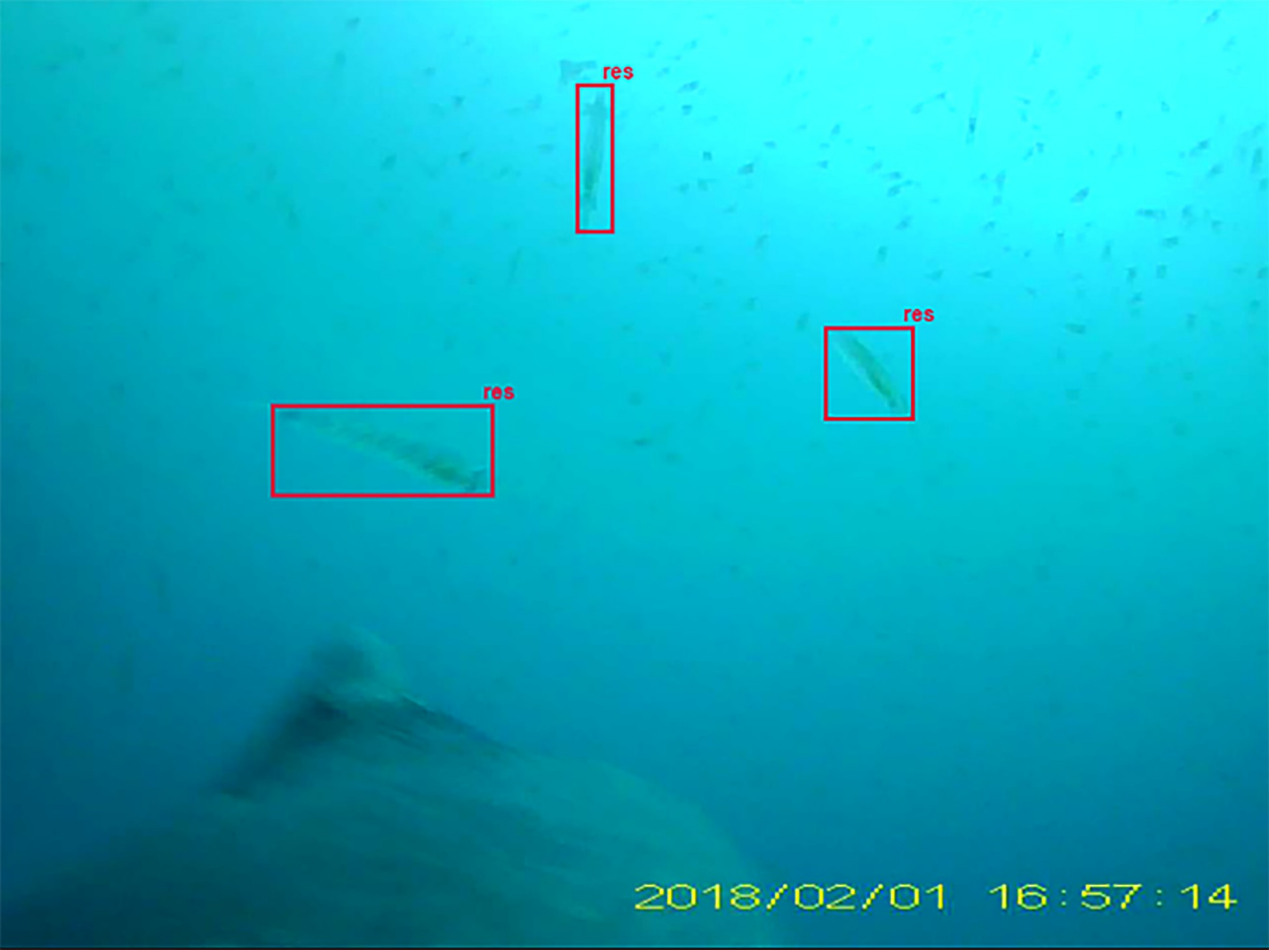
Example of resolvable annotations in an image. Determining the resolvability of krill is dependent upon the contents within each individual frame. Krill within the resolvable range of the camera are apparent; however, smaller targets presumed to be krill are classified as an unresolvable swarm because the distinguishing features of krill (eyes, appendages, body shape) are not visibly distinct.

Krill imaged length at the resolvable range limit was estimated by manually classifying all imaged targets as either resolvable or unresolvable (Fig 4). For resolvable krill, we also annotated whether they presented as axial (when a krill is perpendicular to the camera), partial (when part of the animal was out of the frame), bent, or motion-blurred. Resolvable krill were identified based on distinguishing features such as segmentation of the thoracic region, eyes, carapace, telson, or uropods. In some instances, krill body parts are not visible, but the body shape is distinguishable, and in general this was the strongest indicator that an object was a krill. Unresolvable objects are visually indistinguishable and lack any landmark features or shape attributes that might reliably indicate krill even in the context of a monospecific swarm aggregation.

Note that body orientation will affect measured length in the images. We observed krill in any orientation without apparent tendency for a particular direction relative to the penguin or camera, where krill orientations range from full normal incidence (side, dorsal, or ventral) with projected imaged size as complete length, to axial (head or tail) with projected size representing width. We accounted for this variation in body orientation relative to the camera by assuming that krill present in a uniform distribution of orientation angles from -180 to 180 (Fig 1). As such, the median of imaged lengths projected through a uniform distribution of angles, accounting for non-zero axial length, was 0.707 times the full normal incidence length. This is equivalent to a length projected at +-45 or +-135 degrees from normal incidence. The inverse of this value, 1.414, was used to compensate mean and median imaged lengths (not individual lengths) for the orientation distribution.

**Fig 5:**
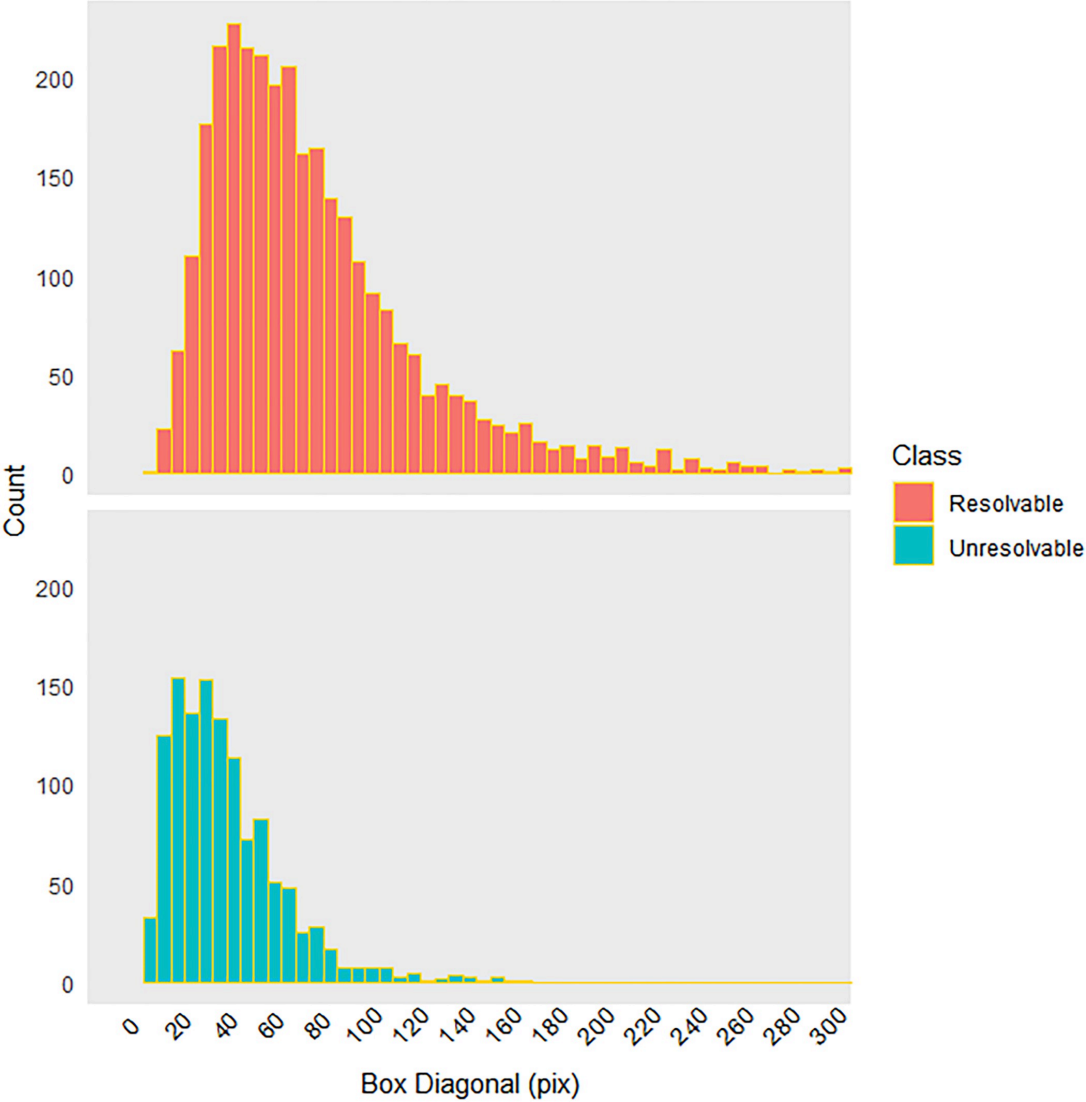
Length frequency distributions (pixel length) for resolvable and unresolvable Antarctic krill (*Euphausia superba*). There is overlap between the lower values of the resolvable krill imaged lengths and the upper values of the unresolvable krill imaged lengths indicating the range of where our resolvable range limit would occur.

Because the images are acquired using a single camera, we do not have a direct measurement of range to imaged objects, therefore we estimate the resolvable range based on the size at the transition from resolvable to unresolvable objects. We assume that resolvable and unresolvable objects generally correspond to objects within and beyond the imaged volume, respectively. Also, because we expect that krill have a narrow size distribution, the size distributions of resolvable and unresolvable objects would be differentiable because of the effect of distance on imaged size (resolvable being closer/large and unresolvable being farther/smaller, in general). The measured image size distributions of unresolvable and resolvable targets are overlapping (Fig 5) and do not indicate a sharp transition or bound on the imaged volume, but they do allow us to estimate the resolvable range.

To estimate resolvable range, we fit a logistic regression model (binary classifier) to imaged sizes of objects assigned to the resolvable (krill) and unresolvable object classes. Size data were excluded for partial krill (along edges), and tightly packed aggregations not individually labeled. The resolvable range was then estimated based on the class division boundary estimated by solving a logistic regression model at p = 0.5 where *L*_boundary_ = 34.4 pixels. The median unresolvable object size, 39.6 pixels, was not much larger than this, and could perhaps be a suitable alternative and more simple to calculate.

To characterize the error and extent of the resolvable range we assumed that all objects with sizes smaller than the logistic regression class size boundary value were unresolvable and that this set of size values represented half the size distribution of objects close to an indistinct boundary marking the limit of the imaged volume. Therefore, we mirrored and duplicated this set of object sizes to produce a symmetrical distribution of size values representing the boundary region and near-boundary objects. This process began by shifting the size boundary set to have a maximum of zero, then we combined that set of values with the absolute value, where we finally shifted back to the boundary value. We did this instead of using the full unresolvable object size distribution (Fig 5) because the unresolvable class included many objects with larger-than-expected sizes that were believed to be krill located within the imaged volume but affected by motion blur strong enough not to allow identification. By strict adherence to the class definitions, these were assigned as unresolvable, and this appeared to have biased the distribution. We fit a normal distribution to the mirrored near-boundary size data and estimated mean and standard deviation.

Resolvable range in imaged units r_res_ (pixels) is estimated as the distance from the camera based on known image dimensions (width: 1,280 pixels, height: 960 pixels) and the camera lens angles (31 by 24°). We assumed that the imaged krill were similar in size to krill obtained from penguin diet samples that were collected [61] and measured [62] during the 2018 field season. Stomach samples were obtained from 20 chick-provisioning penguins in January and February of 2018. The lengths of up to 55 (47 +/- 10, mean +/- 1 sd) intact krill per sample were retrieved and measured for total length to the nearest mm. Resolvable range, width, and height (pixels) are then converted to r_res_ (m) physical units using an imaged-to-physical length conversion factor (mm/pixels) based on the median length (mm) of krill from diet samples divided by the near-boundary imaged length of krill. Imaged volume is calculated as $V=(w*h*r_{res})/3m^{3}$.

### Sensitivity and error

To characterize the sensitivity of our method to uncertainty in identification of a range boundary defining a transition from resolvable to unresolvable targets, we estimated resolvable range (m), imaged volume (m^3^), and krill density (N·m^3^) at a discrete set of imaged sizes representing the near boundary distribution statistics: the mean size at L_boundary_ +- 1 and 2 standard deviations. To characterize the far limit of the volume, we also estimated range, volume, and density using the minimum imaged size of resolvable krill (14.9 pixels) and unresolvable objects (9.9 pixels). To characterize error, we generated a random sample (n = 1000) from a normal distribution with parameters fit to observed near-boundary sizes ~N(34.4, 10.5 pixels), excluding zero and negative values, and estimated the corresponding distributions of resolvable range, imaged volume, and krill density.

### Krill density and biomass

All krill that were classified as resolvable were used to calculate krill numerical density (krill·m^-3^) for the number of resolvable krill per image, divided by our aforementioned estimated imaged volume. A length-weight relationship for krill in the Antarctic Peninsula region [62]: *w* = *aL^b^* (with *L* as total length in mm), *a* = 2.236x10^-6^, and *b* = 3.314, was used to estimate mean krill weight w = 0.54 g, and allowed conversion between estimated krill densities (krill·m^-3^) to biomass density (g·m^-3^).

### Comparison of model predictions to manual annotations

A separate data set with manual annotations of the timing of krill strikes, diving, and surfacing based on previously reported methods [40] was compared to model predictions of frame-level image content. From this, we report summaries of foraging behaviors, including dive and feeding durations, and estimates of swarm thickness (i.e. vertical distance in meters) based on the depth range between the start and end of strikes at krill.

## Results

### Frame-level content classifier

The classifier model was overall 73% accurate in being able to correctly identify frame-level image content. For the krill class performance, metrics indicated an accuracy of 55%, precision of 83% and recall of 63% (Table 2). Performance metrics per class varied. The surface class had particularly high values of 92% accuracy and 99% recall, whereas the penguin presence class had a low value of 15% accuracy and precision. On the other hand, the open water class resulted in 46% accuracy and 67% precision (Table 2).

**Table 2:** Class-specific performance evaluation metrics for a full-frame classification model.

Statistic	Bright-dark	Krill	Surface	Open water	Penguin presence
Accuracy	0.49	0.55	0.92	0.46	0.15
Recall	0.49	0.63	0.99	0.59	0.95
Specificity	0.99	0.95	0.96	0.86	0.89
Precision	0.84	0.83	0.93	0.67	0.15
Negative predicted value	0.96	0.88	1.00	0.82	1.00
Prevalence	0.07	0.26	0.32	0.32	0.02
Balanced Accuracy	0.74	0.79	0.98	0.73	0.92
F-1 score	0.62	0.72	0.96	0.63	0.26

### Krill length and weight

The length of krill measured from diet samples ranged from 31 to 60 mm with a mean length of 43 mm (SD = 4.3 mm) and a median of 42 mm. We assume that these lengths are representative of the physical length distributions of krill observed in images collected in the 2017/2018 field season. Mean weight was estimated as 0.54 g (std. dev = 0.2 g).

### Resolvable range and imaged volume

The classification model predicted that 1,932 of the 27,006 frames (7.2%) contained krill. From these predictions, we selected 124 images (all from December 2018) with minimal motion blur for analysis. Manual classification of resolvability yielded 5,055 krill assigned to resolvability classes, 3,079 resolvable, 154 bent, 193 partial, 12 axial, 125 motion-blurred, and 1,474 unresolvable objects. Imaged lengths ranged from 15 to 766 pixels for resolvable krill, and 10 to 177 pixels for unresolvable objects (Fig 5). The median imaged length of resolvable krill was 72 pixels and the mean imaged length was 85 pixels (sd = 55 pixels), with less than 1% of resolvable krill exceeding 400 pixels. The mean imaged length of unresolvable objects was 45 pixels (sd = 25 pixels, median = 40 pixels).

Using the resolvable-unresolvable class boundary size value resulting from the logistic regression (34.4 pixels), the estimated resolvable range was 1.95 m (Table 3). The quotient of L_boundary_ after compensating for the orientation distribution (L_boundary_/0.707 = 48.7 pixels) and the median of the measured krill (42 mm) from diet samples resulted in a conversion value of 1.16 pixels/mm at the primary boundary. The resolvable range, defined by the object size at the class division boundary, was r_res_ = 1.947 m and the corresponding imaged volume was 0.59 m^3^.

**Table 3:** Summary statistics for each of the boundary regions. As defined, including the size in pixels at the boundary, the resolvable range from the pixel value, imaged width, imaged height, imaged volume, krill density mean, and krill biomass based off of the density mean.

Boundary	Size at boundary (pix)	Res range (mm)	Imaged width (mm)	Imaged height (mm)	Imaged volume (m^3^)	Krill density (krill·m^-3^) (mean)	Krill biomass (g·m^-3^)
size_unres_min	9.9	6774	3839	3839	24.96	1.15	3.86
size_res_min	14.9	4511	2557	2557	7.37	3.90	13.09
**sizeatclassdivision_mean**	**34.4**	**1947**	**1104**	**1104**	**0.59**	**48.47**	**162.70**
sizeatclassdivision_mean-1sd	23.9	2802	1588	1588	1.77	16.26	54.59
sizeatclassdivision_mean+1sd	44.9	1492	846	846	0.27	107.75	361.71
sizeatclassdivision_mean-2sd	13.4	4996	2832	2832	10.02	2.87	9.63
sizeatclassdivision_mean+2sd	55.5	1209	685	685	0.14	202.36	679.34

The object size at the resolvable-unresolvable class boundary estimated by the logistic regression models is L_boundary_ = 34.4 pixels and also represents the mean of the normal distribution fit to near-boundary sizes with sd(L_boundary_) = 10.5 pixels. Upper and lower 67% and 95% size intervals (mean +- 1 and 2 sd) from this fit were [23.9, 44.9] and [13.4, 55.5] pixels.

### Krill density and biomass

**Fig 6:**
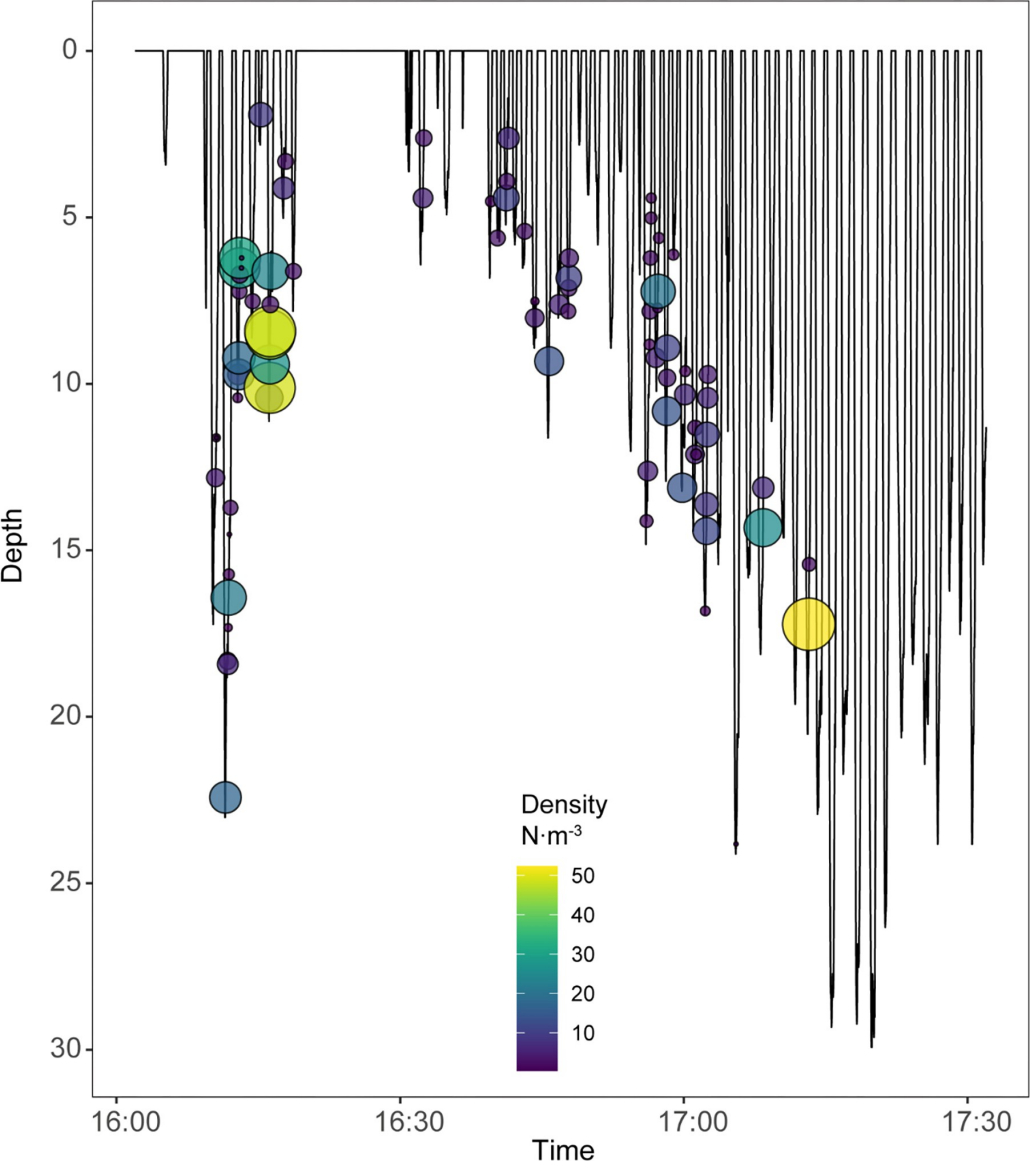
Antarctic krill (*Euphausia superba*) densities per image within the chinstrap penguin’s (*Pygoscelis antarcticus*) dive profile for a 90-minute timespan.

**Fig 7:**
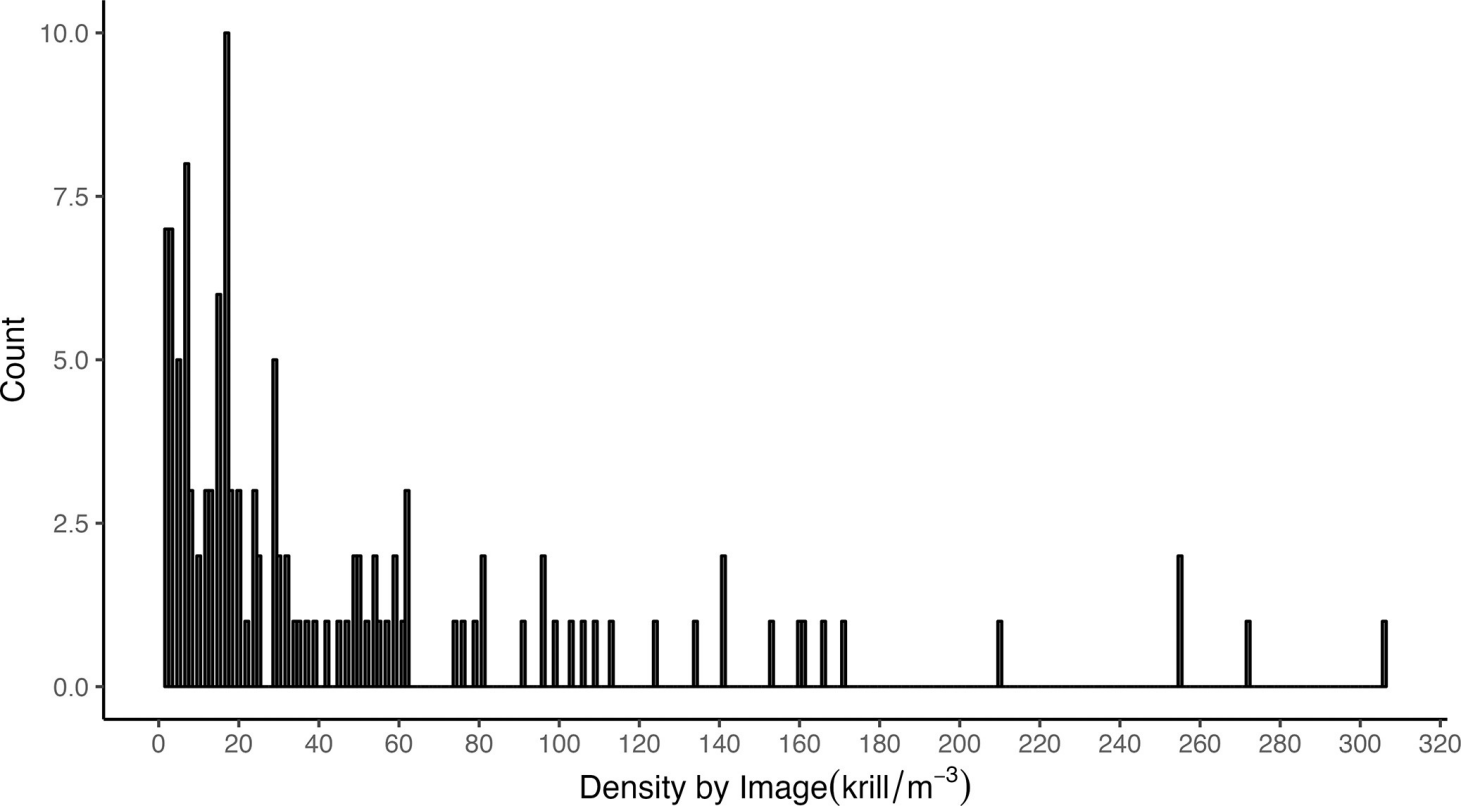
Histogram of estimated Antarctic krill (*Euphausia superba*) densities (n·m^-3^) from 124 krill images predicted by the image classifier. The y-axis represents individual krill counts and the x-axis represents the density values observed from our imagery. Mean krill density was 48 krill·m^-3^ and the standard deviation was estimated as 61 krill·m^-3^.

Krill density in a given image ranged from 2 to 307 krill·m^-3^ over 26 dives (Fig 6). Mean density was 48 krill·m^-3^ (sd = 61 krill·m^-3^) and median krill density was 23.6 krill·m^-3^ (Fig 7). Estimated mean biomass density was 162.7 g·m^-3^.

### Sensitivity and error

**Fig 8:**
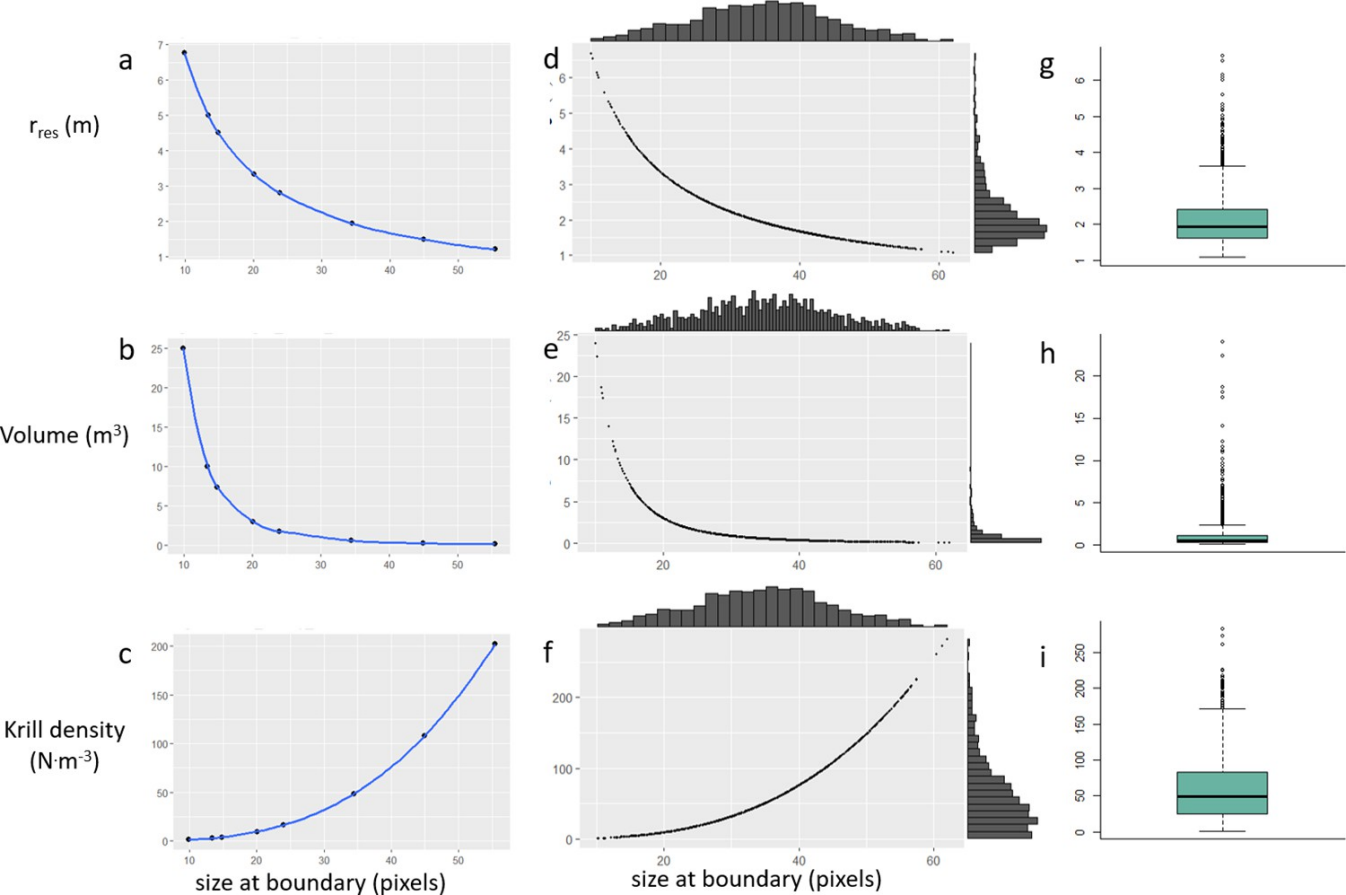
(a) Resolvable range (m), (b) imaged volume (m^3^), and (c), krill density (krill·m^-3^) estimated for the discrete set of boundary values reported in Table 3. (d, e, f) Distributions and (g, h, i) boxplots of resolvable range, imaged volume, and krill density estimated by a simulated sample (n = 1000) of near-boundary object sizes.

Based on the primary size-at-boundary value, L_boundary_ = 34.4 pixels (Table 3; Fig 8) -/+ 1*sd from the mean, 23.9 and 44.9 pixels, the estimated resolvable range and imaged volumes ranged from 2.8 m to 1.5 m and 1.77 m^3^ to 0.27 m^3^, respectively, and mean krill density estimates ranged from 16 to 108 krill·m^-3^. Based on a size-at-bound values -/+ 2*sd from the mean, 13.4 and 55.5 pixels, estimated resolvable range and imaged volumes ranged from 5.0 m to 1.2 m and 10.0 m3 to 0.14 m^3^, respectively, and mean krill density estimates ranged from 3 to 202 krill·m^-3^. The minimum size of resolvable objects was 14.9 pixels and resulted in a resolvable range of 4.51 m, an imaged volume of 7.4 m^3^, and mean mean krill density of 3.9 krill·m^-3^. The minimum size of unresolvable objects was 9.9 pixels and resulted in a resolvable range of 6.77 m and imaged volume of 25.0 m^3^, and mean krill density of 1.2 krill·m^-3^.

Distributions of resolvable range, imaged volume, and krill density resulting from a simulated sample near-object sizes (n = 1000, ~N(34.4, 10.5) pixels), had median values of 1.9 m, 0.58 m^3^, and 49.7 krill·m^-3^ (Fig 8G–8I). Mean values of each sample-estimated distribution were 2.2 m (sd = 0.8 m), 1.2 m^3^ (sd = 2.2 m^3^), 61.1 (sd = 47.9 krill·m^-3^), higher than the medians and suggesting asymmetric distributions (Fig 8D–8F). The 25th and 75th quantile values of these estimates were 1.6 and 2.4 m, 0.34 and 1.15 m^3^, and 25 and 83 krill·m^-3^ (Fig 8G–8I).

### Comparison of model predictions to manual annotations

**Fig 9:**
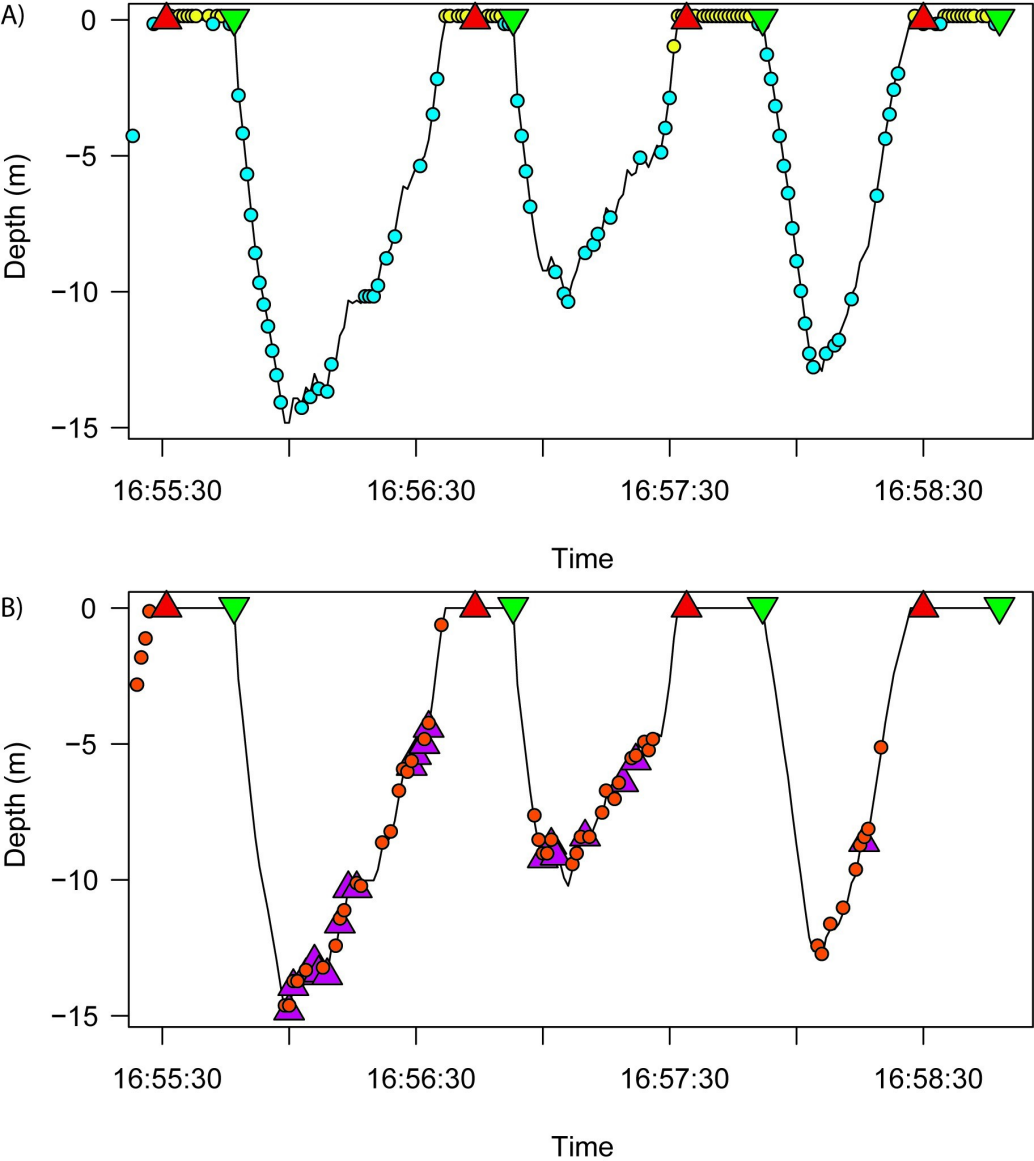
Comparison of the manual annotations and VIAME predictions for a subset of dives. A) Predictions of surface (yellow circles) and underwater (blue circles) frames relative to manual observations of the start (green inverted triangle) and stop (red triangle) of individual dives. B) Predictions of krill frames (orange circles) relative to manual observations of attempted strikes at individual krill (purple triangle).

Model predictions of frame-level content match human annotations of penguin behavior such as surfacing events, underwater activity, transit periods, and prey encounters for a set of three consecutive dive events while foraging (Fig 9). For example, predictions of surface events occurred between the time of dive start and end in manual annotations while predictions for underwater events routinely occurred during dives. In between these surface events, our classifier accurately predicted imagery corresponding with the penguin head being close to the surface of the water, indicating regular breaks to breathe, or resting in place at the surface of the ocean. Our classifier also accurately predicted the transition between immediate descent or ascent (represented by the bright/dark class in our classifier) by flanking surfacing events prior to and after dives. In Fig 9, we illustrate these overlapping characteristics from our classifier model predictions and manual annotations from the video imagery.

## Discussion

We used an image classifier model to automate the identification of frame-level content in animal-borne video imagery and developed a method to estimate prey (Antarctic krill) density as observed in 2D imagery. The results demonstrate that estimating volumetric prey density is possible from 2D video imagery. We note that the classifier model predicted frame-level content consistent with manual observations, highlighting the potential for automated image analysis to quantify a broad suite of penguin foraging behaviors and prey encounter events in video data. Below, we discuss our approach to automating frame-level analysis of video imagery and the several assumptions required for harnessing 2D imaging methods for 3D data collection.

### Automated image analysis

We automated analysis of bio-logging video imagery collected by a chinstrap penguin foraging in the Southern Ocean. Tools such as the video loggers provide researchers with large amounts of data, which have usually been processed manually [40, 63, 64]. As methods for observing marine systems shift to automated systems [65–67] that deploy cameras, the volume of image data collected will increase to levels that exceed the ability and time of analysts to process and analyze manually. In the past, analysts relied on manual annotation [68]. Visually analyzing and manually annotating videos to identify features or events of interest is cumbersome or subject to observer bias, whereas a classifier model improves efficiency and objectivity. Resources like VIAME, that integrate annotation tools with functionality for model training, testing, and application to novel data provide convenient workflows to reduce processing bottlenecks.

Here, our model for frame-level content performed well (Table 2) despite generally low numbers of training images, highlighting the power of the EfficientNet deep learning model to detect gross differences in image content specific to our purpose. We trained the model to characterize video frames based on the environments, behaviors of penguins, and predator-prey interactions. In particular, we were interested primarily in the ability of the model to identify frames that contained krill swarms. The performance evaluation showed reasonably high precision in correctly identifying krill frames (83%). This means that 17% of incorrectly predicted krill imagery (34 out of 198 images) was likely due to a lack in clarity of images affected by motion-blur or faint swarms in the distance. Recall for krill was 63%, which is a key indicator in determining the model’s sensitivity competence. Meanwhile, accuracy for the krill class was only 55%, and is determined by dividing the total number of correct predictions (i.e., TP and TN) divided by the total predictions. The largest discrepancy appeared to be from the krill and open water classes and may have two simple explanations. First, open water comprises most of the image content for these classes. Based on the results of these evaluation metrics, it is fair to assume the model could not differentiate between krill and open water at times. This is reasonable because some of the imagery we used contained large swarms of unresolvable krill in the distance, making it difficult to discern at times. Second, if krill images are affected by motion blur, or if krill targets are at large ranges or have low contrast given ambient lighting they present as small, poorly-resolved targets in the distance and difficult to identify even by human analysts. The model was sufficiently accurate for our purpose to identify the timing of krill encounters and conditions associated with basic diving behavior (Fig 9). Our results highlight the potential for future development of similar models to assess diving and foraging behaviors from video imagery.

### Estimating density from 2D imagery

The process of estimating prey density from 2D animal-borne video imagery requires several assumptions about the size distribution and orientation of targets relative to the camera. With a single lens, we cannot directly measure distance between the camera and individual krill targets. To extrapolate from imaged lengths to real-world lengths, we assumed that all krill observed are equivalent to the median length of krill found in the stomach contents of other penguins. This assumption is reasonable given the narrow size range of krill typically consumed by chinstrap penguins [61] and the consistent tracking of variation in krill sizes by different penguin species over time [69]. Additionally, variation in the orientation of krill relative to the camera can affect the estimates of target lengths in the image. For example, if all krill were observed axially (i.e., only head or tail face the camera), then estimating body length would be impractical. Based on the subset of imagery we used, these extreme orientations were rare. However, most individuals were observed in variable orientations, likely due to escape behaviors and scattering in multiple directions as a predator approached. It was difficult to discern if krill were directly perpendicular to the camera as well, therefore, the variation in orientations necessitated a mechanism to adjust imaged lengths for potential angular shifts. We therefore assumed that krill orientation angles were distributed randomly with a uniform distribution. These assumptions provide the basis to estimate the distance of targets from the lens and construction of 3D information from the video image.

Environmental factors may also affect the estimate of imaged volume, including water column conditions (e.g., visibility, lighting, or turbidity). Water column conditions like visibility, lighting, or turbidity can vary, particularly when imagery is captured at different depths or at different times of the day when solar incidence differs. For example, if krill foraging occurred at greater depths or was recorded near sunset, it is likely that our ability to resolve targets would weaken as light levels fall, effectively reducing the volume of the image. Here, our methodology is limited in that we worked to minimize such effects by focusing on well-lit surface waters <30m in depth (Fig 6) within a short span of foraging activities during midday. Water clarity within these was also similar across the short time span for which video data were available. Our estimate of imaged volume may not be applicable across a broad spectrum of conditions due to the relatively small size of the dataset. This is not a large enough sample size of imagery containing observations from near to far; nor is there enough variation in water column conditions. This method can, nonetheless, address density estimates through assessment of the resolvability of targets in such images.

Another key assumption in our method is how to identify the appropriate cut-off for the size of resolvable krill that represent the limit of the resolvable range. The concept of resolvable range considers that resolvability (or focus) of targets is limited primarily by distance from the camera. In an ideal case without image blur or distortion, resolvable targets would be located near the camera and appear larger while unresolvable targets would be farther away and smaller, assuming that the target size distribution did not vary with range. The resolvable range could then be estimated based on the upper size limit of the unresolvable targets or the lower size limit of resolvable targets. However, in our data there was considerable overlap of the smallest resolvable and unresolvable targets (Fig 5). We assume that many targets are unresolvable for reasons other than distance from the camera, including motion blur. Therefore, we focused our method on the estimated mean from a binary classifier based on a logistic regression fit to sizes of all unresolvable and resolvable objects at defined boundaries between these classes. This normal model fit to near boundary size distributions also provided a probability density function that could be applied to any estimated value of resolvable range, volume, and density.

We estimated density using an imaged volume estimated by resolvable range (essentially depth of field). We use the “resolvability” class data in conjunction with size distribution of objects to estimate the range. While we have an estimate of the range that defines the transition from resolvable to unresolvable objects, we acknowledge the potential for errors of assignment (subjectivity) by one or more analysts. The data and results of the process of assigning targets to a binary scheme demonstrate that there is no clear and strict planar boundary that defines the far edge of the imaged volume. Targets do not transition from being resolvable to unresolvable suddenly over a small change of range. The data reinforce that there is a marginal region, not a plane, where visual characteristics change from sharp and detailed to unidentifiable. We provide estimates of resolvable range, imaged volume, and density for the full range of target sizes that we consider relevant to the process as supported by the data. Our results suggest that the resolvable range is 2m, imaged volume is 0.6 m^3^, and krill density is 50 krill·m^-3^, approximately. The 25th and 75th quantile values of these estimates were within a factor of two times the median values, at 1.6 and 2.4 m, 0.34 and 1.15 m^3^, and 25 and 83 krill·m^-3^, respectively (Fig 8G–8I). Within this transitional region, maybe we could classify targets as marginally resolvable and use their size distribution to estimate the range. Indeed, our initial efforts at target classification included a marginal class, but we determined that it was not feasible to reliably identify objects as marginal. Therefore, we chose the binary scheme, which ultimately allowed us to estimate the resolvable range and imaged volume using the logistic regression classifier, and to characterize the extent of the nebulous boundary.

Similarly, the size distribution of the unresolvable class alone may be enough to estimate the boundary. The sizes of unresolvable objects had a mode of 25, median of 40, mean of 45, and standard deviation of 25 pixels (Fig 5). Given the asymmetry, perhaps the mode or median could serve as alternative estimates of near-boundary object size. The mode of unresolvable object sizes is 9 pixels smaller than the size at boundary estimated from logistic regression. As such, the estimates for range, volume, and density are, respectively, 1.4, 3.4, and 0.4 times the values produced using L_boundary_ = 34.4 pixels (Fig 8 and Table 3). Similarly, the median unresolvable object size is 5 pixels larger and relates to estimates that are 0.9, 1.9, and 1.5 times the same. Unresolvable objects had a wider distribution of sizes than what we used based on assuming that all objects with sizes smaller than the class boundary size from logistic regression were technically unresolvable (and mirroring those to characterize the full marginal region). Therefore, using only the distribution of unresolvable targets is not recommended, not only for this reason, but also because many unresolvable targets are strongly blurred krill at close range within the imaged volume. This also suggests that we adhered to assignment of objects to resolvable-unresolvable classes based on visual characteristics of targets and not context, which is more difficult than it may seem. The size distribution of the resolvable targets overlapped with that of the unresolvable objects, suggesting that some of the resolvable targets were probably truly unresolvable, and also perhaps some smaller detail or context led to similar misassignment in the other direction.

We allowed the classified object size data to determine the boundary despite the likelihood that the data probably contained some errors (e.g. large unresolvable targets that existed in the volume). We did not arbitrarily filter the data because we believed that some errors like this existed. Therefore, the boundary size value based on logistic regression is larger and the estimated resolvable range is smaller than a boundary defined on the smallest resolvable targets, which would ideally be located at the limit of the imaged volume. The steep curves in Fig 5 demonstrate the sensitivity of resolvable range and imaged volume estimates to the changes of the smallest, presumably distant, object sizes. For example, the minimum unresolvable object size (9.9 pixels in length) represents the smallest visually distinguishable objects (presumed to be krill and not bubbles) at the largest range that would be considered to represent an imaged volume encompassing all objects. The resolvable range estimated based on the smallest unresolvable objects was 6.7 m and imaged volume was 25.0 m^3^. The minimum resolvable object size (14.9 pixels in length) represents the smallest identifiable krill. The values of resolvable range and volume associated with this size were 4.5 m and 7.4 m. However, several key points support it is not justified to define a boundary based on the extremes from either class: the probability distribution of near-boundary and unresolvable object sizes; the difficulty for an analyst to distinguish and mark such targets for measurement; and that such small and indistinct objects are not identifiable.

Someone attempting to apply this method to their own imagery should not discount the difficulty in assigning targets to the resolvable and unresolvable classes. There is subjectivity involved, and target details and overall appearance can be strongly affected by factors such as motion blur, irregular illumination, or lighting deficiencies that require the analyst to assign a target to the unresolvable class if they are adhering strictly to the definition. Quantitative methods such as intensity gradients of targets may be applied to classify objects as resolvable/unresolvable, or in focus/not in focus [70], but those gradients would be diminished for objects that are affected by strong motion blur, for instance, leading to the same conclusion as the human visual analyst. Expert annotations are critical to the process, here and in general, for generating datasets that serve as a basis for training more complicated models. Augmented annotations based on intensity gradient metrics for example, would also require careful considerations and perhaps filtering if a trained model is to be trusted. Platforms with moving targets such as this dataset remain a challenge.

We further tested the validity of our biomass estimate relative to verified acoustic survey biomass estimates from the same time period [67]. Currently, there are a lack of studies available to affirm relative thickness of krill swarms actively being foraged by penguins, however, there are some that have estimated swarm vertical thickness [71, 72]. Swarm types 1 and 2 of [72] seemed to represent the swarm types similar to what have been observed by acoustic surveys in our study area [67]. Mean thickness of swarms according to [71] and the type 2 swarms of [72] was 10 m, and mean thickness of the type 1 swarms of [72] was 5 m. Assuming that the penguins in our study dove past the bottom of the swarms before ascending, mean thickness according to the penguins was approximately 7 m (Fig 9). These various krill swarm thicknesses were considered in order to estimate volumetric biomass densities from areal biomass estimates.

The average areal biomass density during January in the area (based on glider acoustic survey [67]) was 52.9 g·m^-2^. Assuming a swarm thickness of either 5 m [71] or 10 m [72], the areal biomass estimate from [67] converts to volumetric biomass densities of 10.57 g·m^-3^ and 5.3 g·m^-3^, respectively. The volumetric biomass density estimated from our imagery (krill counts and estimated imaged volume) was 26.1 g·m^-3^, which is about 2.5 times larger than acoustic survey estimates from the previous year [67] assuming a 5-m thick swarm, and about 5 times the density assuming 10-m thick swarms. If we use the average foraging depths from our study (Fig 9), and assume that swarms were 7 m thick, then the volumetric biomass estimate of [67] was 7.6 g·m^-3^, or 3.4 times less than our image based estimate. Biomass density values from repeated acoustic surveys one month apart in this area differed by a factor of 2 [67]. Given the limitations in vertical swarm thickness, and large acoustic estimate discrepancies between the austral months, our mean volumetric biomass is valid. This confirms the ability in which this methodology can estimate local krill density at a scale that has not yet been observed. Yet, it is important to emphasize our limited study is not representative of general krill population patterns and future efforts should consider utilizing a larger dataset over a larger timescale to discuss broader implications.

## Conclusion

We estimated krill density from an arbitrarily-chosen subset of single camera images collected from the perspective of one penguin. Consequently, we stress that the data reported here are not representative of the krill population as a whole, nor representative of the full range in size and density of krill swarms likely to be encountered during a foraging trip. Rather, we highlight that the method is capable of assessing a wide range of swarm densities that a penguin may encounter. The results provide insight to foraging patterns of individual penguins and their prey availability. For example, krill density encountered by a foraging penguin can change dramatically within and between dives (Fig 6). Such small-scale variation in prey density on short time and space scales suggests a high degree of patchiness in krill swarms that likely impacts foraging success and effort by air breathing predators like penguins.

The limitations of single image and absence of depth data has required us to estimate range and volume based on target object characteristics with possibly much more uncertainty than if we could measure depth directly. Perhaps there are other options for cameras and lenses that could achieve a sharper boundary division, a more discrete depth of field, and more importantly, direct measurement of distance (range) to objects. Range and target size estimates could be accomplished by using a depth sensor next to the camera (as in RGB-D) or stereo camera systems. A system that could create an accurate, dense 3D reconstruction of the scene at high speed and simultaneously track each target to enable identification and measurement would be valuable. Such a system would facilitate the ability of more detailed quantification of predator-prey interactions. However, we are not aware of any available systems that are small enough, with a small enough mass, to be borne by small marine organisms. Motion blur, caused by simultaneous high-speed motion and rapid changes of direction by the predator and prey, is a big problem which might be addressed by using a high intensity short duration strobe, but in shallow, clear water during daytime, the efficacy of such a strobe may be diminished. Therefore, very high-speed image acquisition rates and very short exposure durations may be preferable. We would encourage the development of such hardware that could be borne by small marine animals like penguins. Until then, the method we describe may be used to estimate imaged volume and prey density from single cameras.

Future work to examine the links between estimates of prey density with consumption rates, foraging behavior (e.g., decisions to continue foraging locally or transit to new foraging areas), and the energetic expenses associated with those decisions are likely to yield important insight in how real-time variation in prey density affects predators. Additionally, prey density data from predator-borne systems may provide information important to managing krill fisheries, such as how well the density estimated from surveys using ships or autonomous vehicles [70] represents the prey directly available to predators. Furthermore, with a changing climate, data such as this may provide a first-hand predator perspective on changes in prey availability. The methods used here to estimate volumetric density from 2D imagery should be generally applicable to other open-water systems with swarming prey.
